# Cognitive behavioral therapy for anxiety and depression in cancer survivors: a meta-analysis

**DOI:** 10.1038/s41598-022-25068-7

**Published:** 2022-12-12

**Authors:** Lemeng Zhang, Xiaohong Liu, Fei Tong, Ran Zou, Wanglian Peng, Hui Yang, Feng Liu, Desong Yang, Xufen Huang, Lili Yi, Minni Wen, Ling Jiang

**Affiliations:** 1 Thoracic Medicine Department 1, Hunan Cancer Hospital/The Affiliated Cancer Hospital of Xiangya School of Medicine, Central South University, Hunan Province, Changsha, 410013 People’s Republic of China; 2 Department of Clinical Spiritual Care, Hunan Cancer Hospital/The Affiliated Cancer Hospital of Xiangya School of Medicine, Central South University, Hunan Province, Tongzipo Rd 283#, Yuelu District, Changsha, 410013 People’s Republic of China; 3 Department of Radiation Oncology, Hunan Cancer Hospital/The Affiliated Cancer Hospital of Xiangya School of Medicine, Central South University, Hunan Province, Changsha, 410013 People’s Republic of China; 4 Department of Thoracic Surgery, Hunan Cancer Hospital/The Affiliated Cancer Hospital of Xiangya School of Medicine, Central South University, Hunan Province, Changsha, 410013 People’s Republic of China

**Keywords:** Cancer, Psychology, Health care, Oncology

## Abstract

This study aimed to investigate the effects of cognitive behavioral therapy (CBT) on anxiety and depression in cancer survivors. The PubMed, Embase, PsycINFO, and Cochrane Library databases were searched. Randomized controlled trials that evaluated the effects of CBT in cancer survivors were included. The standardized mean difference (SMD) was used as an effect size indicator. Fifteen studies were included. For the depression score, the pooled results of the random effects model were as follows: pre-treatment versus post-treatment, SMD (95% confidence interval [CI]) = 0.88 (0.46, 1.29), *P* < 0.001; pre-treatment versus 3-month follow-up, 0.83 (0.09, 1.76), *P* = 0.08; pre-treatment versus 6-month follow-up, 0.92 (0.27, 1.58), *P* = 0.006; and pre-treatment versus 12-month follow-up, 0.21 (− 0.28, 0.70), *P* = 0.40. For the anxiety score, the pooled results of the random effects model were as follows: pre-treatment versus post-treatment, 0.97 (0.58, 1.36), *P* < 0.001; pre-treatment versus 3-month follow-up, 1.45 (− 0.82, 3.72), *P* = 0.21; and pre-treatment versus 6-month follow-up, 1.00 (0.17, 1.83), *P* = 0.02). The pooled result of the fixed effects model for the comparison between pre-treatment and the 12-month follow-up was 0.10 (− 0.16, 0.35; *P* = 0.45). The subgroup analysis revealed that the geographical location, treatment time and treatment form were not sources of significant heterogeneity. CBT significantly improved the depression and anxiety scores of the cancer survivors; such improvement was maintained until the 6-month follow-up. These findings support recommendations for the use of CBT in survivors of cancer.

## Introduction

The concept of cancer survivors was first proposed by Fitzhugh Mullan, a physician diagnosed with cancer^1^. According to the National Coalition for Cancer Survivorship, “an individual is considered to be a cancer survivor from the time of diagnosis through the balance of his or her life.” This definition includes family members, caregivers, and friends because survivorship experience also affects them^2^. With the promotion of cancer screening and improvements in treatment, the survival rate of patients with cancer continues to improve. This leads to a dramatic increase in the number of survivors over the past few decades^3,4^. As reported by the National Cancer Center based on data from 17 cancer registries in China, the 5-year survival rates for 26 types of cancer increased from 30.9 to 40.5% between 2003 and 2015^5^. In the United States, the number of cancer survivors increased from approximately 3 million in 1971 to nearly 15.5 million in 2016^6–8^. This number is expected to reach more than 26 million by 2040^6,7^. Owing to the prolonged survival period after treatment, efforts need to be made to improve the quality of life and survival status of cancer survivors.

Cancer survivors often face physical, psychological, and psychosocial challenges that extend into long-term survivorship^9,10^. It has been reported that they are prone to experiencing fatigue, sleep disorders, chronic pain, fear of recurrence, anxiety, and depression, which not only disrupt the quality of life and return to usual activities but can also be barriers to engaging in survivorship care^11–13^. Among these psychosocial challenges, psychological problems, such as depression and anxiety require early identification, because they are often under-diagnosed and under-treated^14^. Some cohort studies have shown that cancer survivors report higher rates of anxiety and depression than individuals without a history of cancer^15,16^. Depression is reported in approximately 8–33% of patients with cancer and anxiety in approximately 17–23%^17,18^. Depression is associated with poor adherence to cancer treatment and poor survival^19,20^. Additionally, it is detrimental to quality of life and is correlated with a two-fold increase in the risk of all-cause death among cancer survivors^21^. Psychological anxiety makes patients irritable, unable to concentrate, negative, and very pessimistic, all of which can decrease their quality of life^22^. For cancer survivors, these psychological disorders not only interfere with quality of life but can also become barriers to engaging in survivorship care. This is especially for women, adolescents, and young individuals, because they are particularly at risk for mood disturbances^23^. Seriously, these disorders are difficult to alleviate with drugs, leaving the needs of cancer survivors for improved quality of life, especially their psychosocial needs, far unmet. Health-related quality of life is a multidimensional construct that encompasses physical functioning as well as psychosocial aspects of emotional and social functioning. There has been a paradigm shift in health service delivery to a more holistic approach, which considers quality of life and overall functioning^24^.

Several studies have reported that psychosocial interventions can effectively treat these distressing emotions, with cognitive behavioral therapy (CBT) being the most frequently used approach^25^. The term CBT describes a group of psychotherapeutic techniques that treat psychological distress and maladaptive behaviors by changing cognitions and behaviors^26^. CBT describes a hybrid of strategies to facilitate cognitive, behavioral, emotional and social change. The interventions include the teaching of social skills through role playing, problem solving techniques, coping skills, examining alternative ways of perception, and engagement in verbally mediated self-control^27^. According to CBT, the emotions and behaviors of individuals are determinants of their cognitive processes. Once cognitive defects are corrected, negative emotions and behaviors improve. As a result, CBT aims to modify cancer survivors’ wrong cognition into a more rational manner of thinking, helping them gain a sense of control over the disease and increasing their confidence in fighting it^28^. Furthermore, CBT has been traditionally used for patients with mental health disorders, such as depression and anxiety^29^. Many randomized controlled trials (RCTs) have examined the effects of CBT on anxiety and depression among cancer survivors. However, the results are inconsistent and not comprehensive because of the wide variations in sample sizes, ethnicities, and outcome assessment methods used.

In this study, a meta-analysis of RCTs was conducted to comprehensively evaluate the effect of CBT on anxiety and depression in cancer survivors through a dynamic follow-up from 3 to 12 months.

## Methods

The meta-analysis procedure was performed in accordance with the Preferred Reporting Items for Systematic Reviews and Meta-Analysis (PRISMA) statement guidelines^30^. As this study analyzed data from previously published studies, ethical ratification was not required. Considering that this study is a meta-analysis study using the existing peer-reviewed literature,and no human/animal patients were directly involved in the study, receiving their con-sent to participate or consent to publish was not considered as necessary.

### Search strategy

According to the predefined search strategy, we identified appropriate literature using the following electronic databases: PubMed, PsycINFO, Embase, and Cochrane Library. The search keywords included “cognitive behavioral therapy,” “cognitive behavior therapy,” “neoplasms,” “cancer,” “anxiety,” and “depression.” Keywords in the same category were combined with “OR” and those in different categories with “AND.” Subject terms and free words were searched in combination, and the retrieval method was adjusted according to database characteristics. The retrieval steps for the PubMed database are presented in Supplementary table 1. We focused on articles published up to May 23, 2022, without language restrictions. Additionally, the references of relevant reviews and the included literature were searched for eligible studies.

### Inclusion and exclusion criteria for study selection

The inclusion criteria for the studies were as follows: (1) participant: cancer survivors (patients with cancer who had completed treatment, except for targeted treatments or hormonal treatments); (2) variable compared: differences in the effects of CBT and treatment as usual (TAU) on depression and anxiety in patients with cancer; and (3) study type: RCT.

The exclusion criteria were as follows: (1) non-literary research, such as review and meeting abstracts; (2) third-generation CBT, such as mindfulness-based cognitive therapy and acceptance and commitment therapy; (3) patients receiving or preparing to receive standard treatments, such as surgery, radiation, chemotherapy, or immunotherapy; and (4) repeated publications or multiple articles with the same data (only the article with the most complete research information was retained).

### Data extraction and quality assessment

Two reviewers independently completed literature screening. After obtainment of the included literature, information on the first author, publication year, country, basic participant characteristics (sample size, sex, and age), cancer type and stage, follow-up time, intervention period, and study outcome was independently extracted according to the pre-designed table. After the data extraction, the two reviewers exchanged the tables, and disagreements were resolved via discussion. The quality of the RCTs was assessed using the Cochrane Collaboration’s tool^31^.

### Statistical analysis

The standardized mean difference (SMD) and 95% confidence interval (CI) were used as the effect size indicators to evaluate the differences in the anxiety and depression scores between post-treatment and the 3/6/12-month follow-up. Cochran’s Q test and I^2^ test were used for heterogeneity testing^32^. *P* < 0.05 or I^2^ > 50% indicated significant heterogeneity, and the random effects model was used for the data analysis. Random-effects model attempted to generalize findings beyond the included studies by assuming that the selected studies are random samples from a larger population^33^. *P* ≥ 0.05 or I^2^ ≤ 50% indicated non-significant heterogeneity, and the fixed effects model was applied for the meta-analysis. Fixed-effect models assume that the population effect sizes are the same for all studies^33^. Subgroup analysis was performed according to the geographical location and treatment time. The effect of a single study on the meta-analysis was evaluated using a one-by-one exclusion method^34^. Publication bias was evaluated using the Egger test^35^. When significant publication bias existed, the stability of the combined results was assessed using the trim-and-fill method^36^. All statistical analyses were performed using the Stata 12.0 and RevMan 5.3 software.

## Results

### Literature search

**Figure 1:**
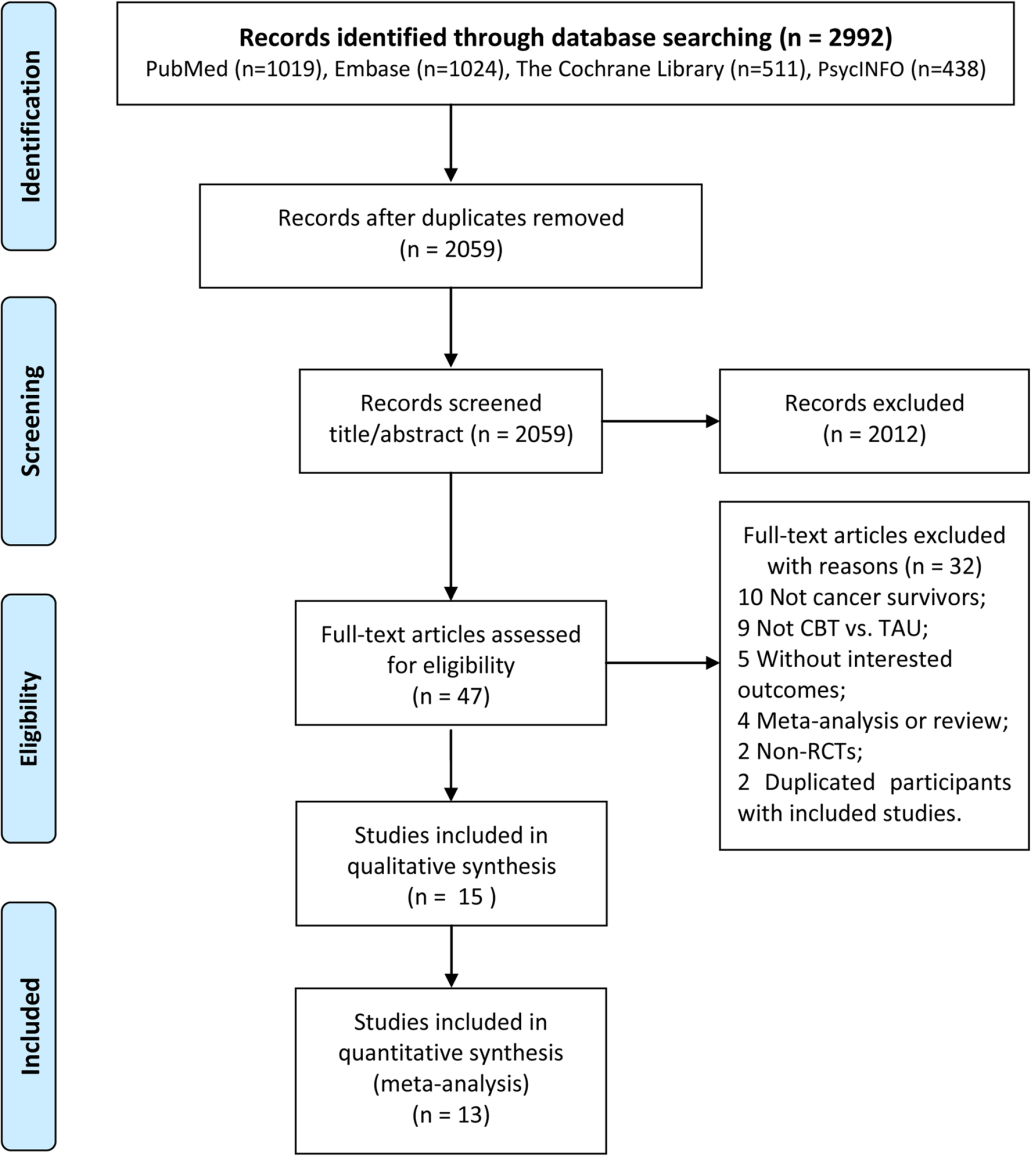
Selection process for the trials included in the meta-analysis. *CBT* cognitive behavioral therapy, *TAU* treatment as usual, *RCT* randomized controlled trial.

The literature retrieval results and screening processes are presented in Fig. 1. A total of 2992 articles were retrieved from the electronic databases (1019 from PubMed, 1024 from Embase, 511 from the Cochrane Library, and 438 from PsycINFO) in this meta-analysis. After duplicate elimination, 2059 articles remained. Thereafter, 2012 articles were further removed by browsing the titles and abstracts. Finally, 15 articles were included after full-text reading, including 13 quantitative analyses^37–49^ and 2 qualitative analyses.

### Study characteristics and quality assessment

The publication dates of the 15 articles ranged from 2003 to 2022. These studies were conducted in China, the United Kingdom, South Korea, Iran, the United States, and Canada. The sample size ranged from 29 to 294, with 1979 cases. Of the included articles, seven reported on patients with breast cancer^37,38,42–44,50,51^, one on patients with melanoma^47^, five on patients with mixed cancers^39,46,48,52,53^, one on patients with laryngeal squamous cell carcinoma^49^ and one on patients with head and neck cancer^54^. The average age of the participants ranged from 37.45 to 59.7 years. The CBT intervention period was 2–12 weeks, and the follow-up period was within 12 months after the intervention. The rating scales used for anxiety and depression are listed in Table 1.

**Table 1:** Characteristics of the included studies.

Study (area)	Type of cancer	Stage	n, M/F	Treatment time	Follow-up	Groups	Case	Age, years	M/F	Outcomes	Intervention
**Quantitative analysis**											
Fenlon, D 2020 (UK)	Breast	NR	127, 0/127	6 weeks	6 months	CBT	61	53.5 ± 9.78	0/61	PHQ, GAD-7	Weekly group CBT sessions, lasting 90 min, for 6 weeks
						TAU	66	55.2 ± 10.19	0/66		
Groarke, A 2013 (Ireland)	Breast	Any	179, 0/179	5 weeks	12 months	CBT	87	53.30 ± 9.86	0/87	HADS-D, HADS-A	In groups of 8–12 for 3 h per week
						TAU	92	54.10 ± 10.62	0/92		
Ham, K 2019 (South Korea)	Mixed	I-IV	42, 6/36	10 weeks	NR	CBT	21	41.90 ± 11.30	3/18	DAS, STAI	One session per day for a total of 10 weeks at home
						TAU	21	47.10 ± 11.19	3/18		
Jelvehzadeh, F 2022 (Iran)	Breast	Any	48, 0/48	8 weeks	3 months	CBT	24	49.40 ± 7.22	0/24	DASS-21	Three groups of 8 were formed and 8 sessions were held for each group
						TAU	24	47.94 ± 6.99	0/24		
Murphy, MJ 2020 (Australia)	Mixed	early	114, 13/101	16 weeks	3 months	CBT	53	53.28 ± 9.22	7/46	HADS-D, HADS-A	Online self-managed but clinician supervised, 16-week, 8-lesson program
						TAU	61	53.30 ± 10.09	6/55		
Qiu, J 2013 (China)	Breast	0-IV	62, 0/62	10 weeks	6 months	CBT	31	51.68 ± 5.95	0/31	HAMD, SAS	Treatment protocol-guided group, weekly for 10 two-hour sessions
						TAU	31	49.58 ± 8.03	0/31		
Savard, J 2005 (Canada)	Breast	I-III	58, 0/58	8 weeks	12 months	CBT	27	54.81 ± 7.01	0/27	HADS-D, HADS-A	Eight weekly sessions of 90 min, offered in groups of four to six patients
						TAU	30	53.37 ± 7.72	0/30		
Savard, J 2006 (Canada)	Breast	metastatic	45, 0/37	8 weeks	6 months	CBT	21	51.47 ± 8.05	0/21	HADS-D, HADS-A	Eight weekly sessions of 60 to 90 min, individually
						TAU	16	51.66 ± 8.62	0/16		
Serfaty, M 2019 (UK)	Mixed	Any	230, 78/152	12 weeks	3 months	CBT	115	59.5 ± 10.3	41/74	BDI-2	Twelve weekly sessions of individual CBT delivered
						TAU	115	59.5 ± 12.4	37/78		
Sheikhzadeh, M 2021 (Iran)	Mixed	Any	39, 7/32	8 weeks	NR	CBT	19	40.10 (mean)	1/18	BDI, BAI	Eight weekly 90-min sessions were held in a room of hospital
						TAU	20	37.45 (mean)	6/14		
Trask, PC 2003 (USA)	Melanoma	0-III	48, 14/34	4 weeks	6 months	CBT	25	56.2 (30–92)	8/17	STAI	Three 50-min weekly group sessions for 4 weeks
						TAU	23	51.0 (22–71)	6/17		
van de Wal, M 2017 (Netherland)	Mixed	NR	88, 41/47	8 weeks	NR	CBT	45	58.0 ± 11.3	21/24	HADS-D, HADS-A	Five individual 1 h session, with three 15-min e-consultations
						TAU	43	59.7 ± 10.0	20/23		
Yang, Y 2022 (China)	LSCC	NR	80, 66/14	2 weeks	NR	CBT	40	51.05 ± 3.66	34/6	SAI, PHQ-9	Five sessions, and each session took about 20 min, individually
						TAU	40	51.13 ± 3.52	32/8		
**Qualitative analysis**											
Duffy, SA 2006 (USA)	HNC	Any	184, 155/29	NR	6 months	CBT	93	56 ± 10.8	72/21	GDSSF	9–11 sessions of CBT telephone counseling
						TAU	91	58 ± 8.9	83/8		
Savard, J 2014 (Canada)	Breast	0–III	242, 0/242	6 weeks	6 months	CBT	81	52.6 ± 8.9	0/81	HADS-D, HADS-A	Six weekly, individual treatment sessions of approximately 50 min
						TAU	81	55.4 ± 8.8	0/81		

F, female; M, male; NR, not reported; HNC, Head and Neck Cancer; LSCC, laryngeal squamous cell carcinoma; GDSSF, Geriatric Depression Scale-Short Form; GAD-7, general anxiety disorder; PHQ, patient health questionnaire; STAI, State-Trait Anxiety Inventory scores; DASS-21, The Depression Anxiety Stress Scales 21; HAMD, 17-Item Hamilton Depression Rating Scale; HAMA, Hamilton anxiety scale; SAS, Self-Rating Anxiety Scale; BDI-2, Beck Depression Inventory, version 2; BAI, Beck Anxiety Inventory; SAI, Spielberger State Anxiety Inventory; DAS, Dysfunctional Attitudes Scale.

The methodological quality assessment results of the included articles are shown in Supplementary Fig. 1A and B. Bias mainly included performance and detection biases. The bias level of the included studies was uncertain, and the methodological quality was moderate.

### Meta-analysis

**Figure 2:**
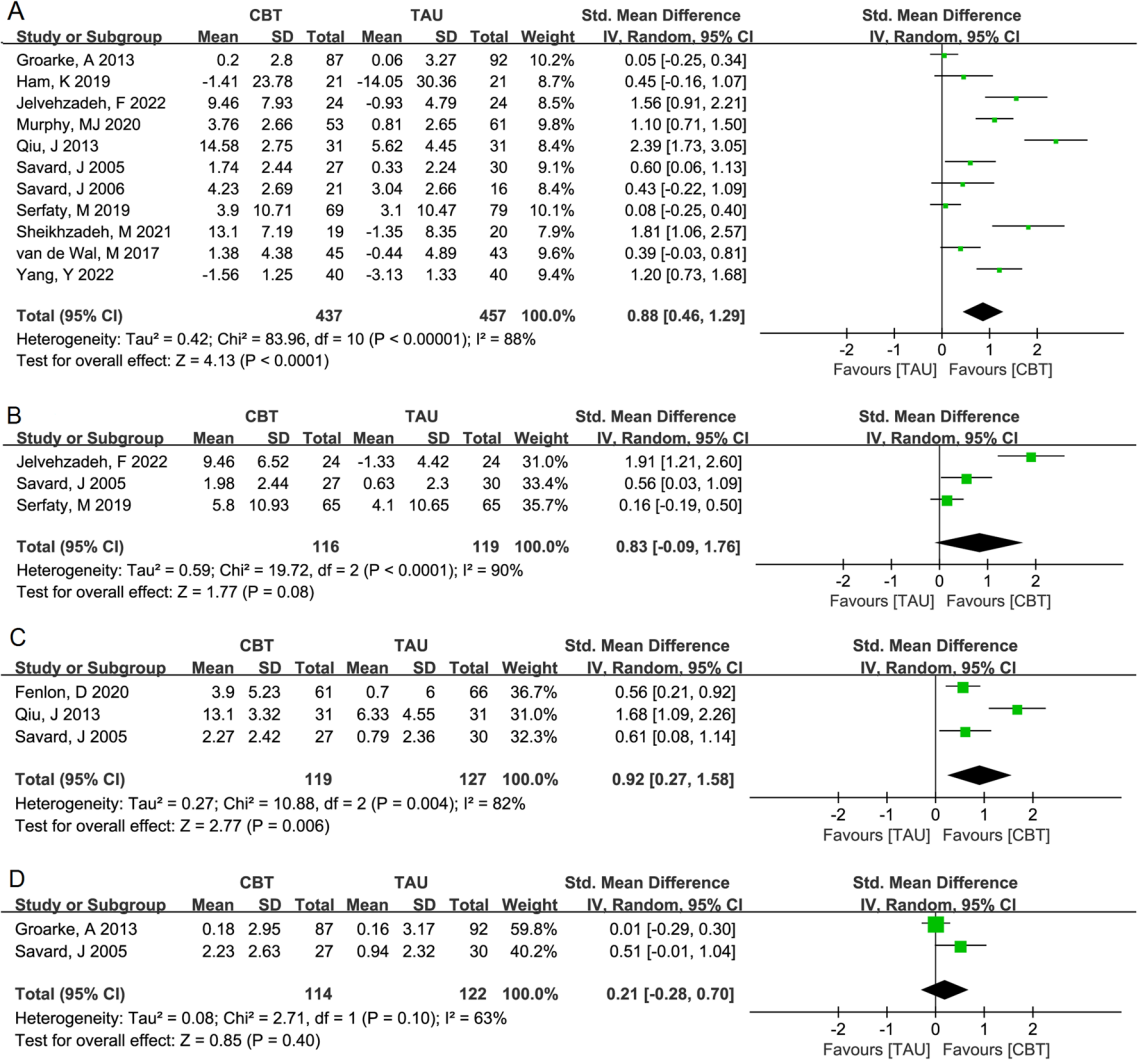
Forest plot of the random effects model meta-analysis of the comparison of the change values of the depression scores between CBT and TAU: (**A**) pre-treatment versus post-treatment, (**B**) pre-treatment versus 3-month follow-up, (**C**) pre-treatment versus 6-month follow-up, and (**D**) pre-treatment versus 12-month follow-up. *CBT* cognitive behavioral therapy, *TAU* treatment as usual.

For the depression score, the change values between CBT and TAU in the four outcome indicators (A, pre-treatment vs. post-treatment; B, pre-treatment vs. 3-month follow-up; C, pre-treatment vs. 6-month follow-up; D, pre-treatment vs. 12-month follow-up) showed significant heterogeneity among the included articles (I^2^ > 50%, *P* < 0.05). The pooled results of the random effects model were as follows: pre-treatment versus post-treatment, SMD (95% CI) 0.88 (0.46, 1.29), *P* < 0.001 (Fig. 2A); pre-treatment versus 3-month follow-up, 0.83 (0.09, 1.76), *P* = 0.08 (Fig. 2B); pre-treatment versus 6-month follow-up, 0.92 (0.27, 1.58), *P* = 0.006 (Fig. 2C); and pre-treatment versus 12-month follow-up, 0.21 (− 0.28, 0.70), *P* = 0.40 (Fig. 2D). The pooled results suggested that CBT significantly improved the depression scores of the cancer survivors after the intervention and at the 6-month follow-up. However, there was no obvious improvement in the depression scores at the 12-month follow-up.

**Figure 3:**
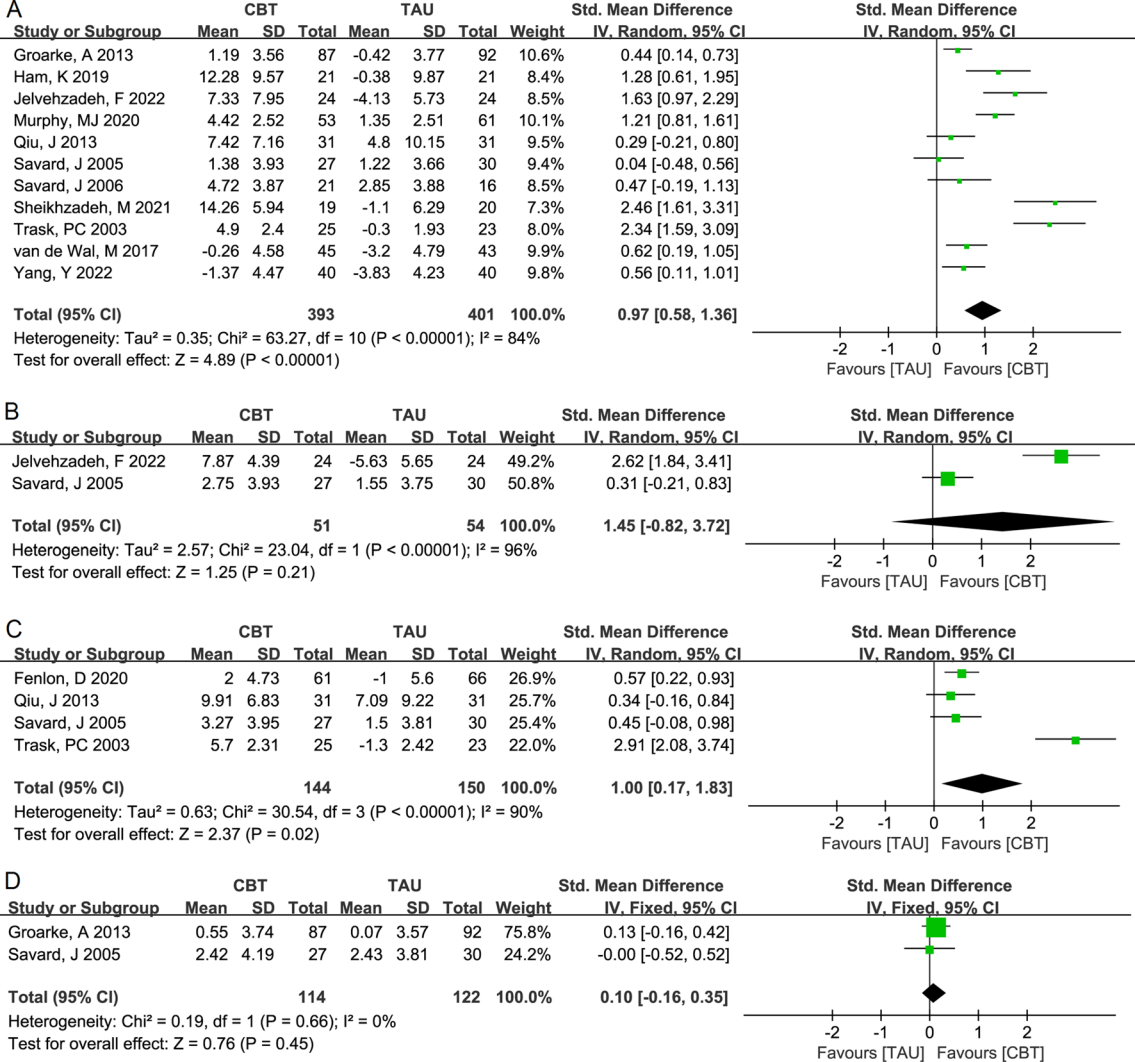
Forest plot of the random effects model meta-analysis of the comparison of the change values of the anxiety scores between CBT and TAU: (**A**) pre-treatment versus post-treatment, (**B**) pre-treatment versus 3-month follow-up, and (**C**) pre-treatment versus 6-month follow-up, and the fixed effects model meta-analysis of the comparison of the change values obtained (**D**) pre-treatment and at the 12-month follow-up. *CBT* cognitive behavioral therapy, *TAU* treatment as usual.

For the anxiety score, the change values between CBT and TAU in the comparisons of pre-treatment with post-treatment, pre-treatment with the 3-month follow-up, and pre-treatment with the 6-month follow-up showed significant heterogeneity among the included articles (I^2^ > 50%, *P* < 0.05). The pooled results of the random effect models were as follows: pre-treatment versus post-treatment, SMD (95% CI) = 0.97 (0.58, 1.36), *P* < 0.0001 (Fig. 3A); pre-treatment versus 3-month follow-up, 1.45 (− 0.82, 3.72), *P* = 0.21 (Fig. 3B); and pre-treatment versus 6-month follow-up, 1.00 (0.17, 1.83), *P* = 0.02 (Fig. 3C). The included articles comparing pre-treatment with the 12-month follow-up showed no significant heterogeneity, and the pooled result of the fixed effects model was SMD (95% CI) = 0.10 (− 0.16, 0.35), *P* = 0.45 (Fig. 3D). The pooled results suggested that CBT also significantly improved the anxiety scores of the patients with cancer after intervention and at the 6-month follow-up. Similarly, there was no significant improvement in the anxiety scores at the 12-month follow-up.

### Subgroup analysis

Since the number of included studies that conducted 3-, 6-, and 12-month follow-ups was fewer than five, this meta-analysis only performed subgroup analysis on the changes post-treatment (Table 2). For the depression scores in the subgroup analysis according to the geographical location, the pooled results of the European subgroup were not significant (SMD [95% CI] = 0.13 [− 0.06, 0.32], *P* = 0.19), whereas the combined effect values of the other subgroups were significant (American: SMD [95% CI] = 0.53 [0.12, 0.95], *P* = 0.01; Asian: 1.47 [0.85, 2.09], *P* < 0.00001; Oceanian: 1.10 [0.71, 1.50], *P* < 0.00001) (Supplementary Fig. 2A). In the subgroup analysis according to the treatment time, the pooled results of the less than or equal to 6 weeks subgroup were not significant (SMD [95% CI] = 0.61 [− 0.53, 1.74], *P* = 0.29), while the combined effect values of the more than 6 weeks subgroup were significant (SMD [95% CI] = 0.95 [0.47, 1.43], *P* = 0.0001) (Supplementary Fig. 2B). For the subgroup analysis of treatment form, the pooled results of group therapy and individual therapy were statistically significant (*P* < 0.05, Supplementary Fig. 2C).

**Table 2:** Results of subgroup analyses.

Outcomes	No. of study	SMD (95%CI)	*P* value	Heterogeneity test	
				I^2^ (%)	P_H_
**Depression**					
Change of Post-treatment					
Overall	11	0.88 (0.46, 1.29)	< 0.0001	88	< 0.00001
Group therapy					
Yes	5	1.25 (0.32, 2.18)	0.008	93	< 0.00001
No	6	0.61 (0.20, 1.02)	0.003	79	0.0002
Area					
American	2	0.53 (0.12, 0.95)	0.01	0	0.71
European	3	0.13 (− 0.06, 0.32)	0.19	0	0.39
Asian	5	1.47 (0.85, 2.09)	< 0.00001	80	0.0006
Oceania	1	1.10 (0.71, 1.50)	< 0.00001	NA	NA
Treatment time					
Less or equal than 6 weeks	2	0.61 (− 0.53, 1.74)	0.29	94	< 0.0001
More than 6 weeks	9	0.95 (0.47, 1.43)	0.0001	87	< 0.00001
**Anxiety**					
Change of Post-treatment					
Overall	11	0.97 (0.58, 1.36)	< 0.00001	84	< 0.00001
Group therapy					
Yes	6	1.14 (0.42, 1.87)	0.002	91	< 0.00001
No	5	0.83 (0.50, 1.16)	< 0.00001	54	0.07
Area					
American	3	0.93 (− 0.37, 2.23)	0.16	92	< 0.00001
European	2	0.50 (0.25, 0.74)	< 0.0001	0	0.49
Asian	5	1.19 (0.50, 1.88)	0.0007	85	0.0001
Oceania	1	1.21 (0.81, 1.61)	< 0.00001	NA	NA
Treatment time					
Less or equal than 6 weeks	3	1.04 (0.16, 1.93)	0.02	91	< 0.0001
More than 6 weeks	8	0.95 (0.48, 1.42)	< 0.0001	83	< 0.00001

CI, confidence interval; NA, not available; SMD, Standardized mean difference.

Similarly, for the anxiety scores in the subgroup analysis according to the geographical location, the pooled results of the American subgroups had no significant difference (*P* > 0.05), while those of the other subgroups had a significant difference (European: SMD [95% CI] = 0.50 [0.25, 0.74], *P* < 0.00001; Asian: 1.19 [0.50, 1.88], *P* = 0.0007; Oceanian: 1.21 [0.81, 1.61], *P* < 0.00001) (Supplementary Fig. 3A). In the subgroup analysis according to the treatment time, the pooled results of both the less than or equal to 6 weeks subgroup (SMD [95% CI] = 1.04 [0.16, 1.93], *P* = 0.02) and more than 6 weeks subgroup (0.95 [0.48, 1.42], *P* < 0.0001) were significantly different (Supplementary Fig. 3B). The pooled results of group therapy and individual therapy were statistically significant (*P* < 0.05, Supplementary Fig. 3). In addition, the subgroup analysis showed that the geographical location, treatment time and treatment form were not sources of significant heterogeneity.

### Sensitivity analysis and publication bias test

Only two studies reported the anxiety scores at the 3-month follow-up and depression and anxiety scores at the 12-month follow-up, making them unsuitable for the sensitivity analysis or publication bias test. The analysis results for the depression and anxiety scores at the other time points are summarized in Table 3. The sensitivity analysis revealed that the intervention effect of CBT was stable at post-treatment, the 3-month follow-up, and the 6-month follow-up. For post-treatment, the SMD (95% CI) of the pooled results changed from 0.73 (0.36, 1.09) to 0.97 (0.54, 1.41); for the 3-month follow-up, from 0.31 (− 0.08, 0.69) to 1.00 (− 0.71, 2.71); and for the 6-month follow-up, from 0.58 (0.28, 0.87) to 1.13 (0.09, 2.18). The sensitivity analysis showed that the pooled results were not significantly affected by a single study. For the anxiety scores, the results were stable at post-treatment, with the SMD (95% CI) changing from 0.84 (0.48, 1.19) to 1.06 (0.66, 1.46). However, the results at the 6-month follow-up were unstable, with the SMD (95% CI) changing from 1.19 (− 0.15, 2.53) to 1.25 (0.08, 2.42).

**Table 3:** Outcomes of the sensitivity analysis and test of publication bias.

Outcomes	No. of studies	Sensitivity analysis	Egger’ s test	
		SMDs (95% CI)	Robust	*P* value
**Depression**				
Change of Post-treatment	11	0.73 (0.36, 1.09) to 0.97 (0.54, 1.41)	Yes	0.011
Change of 3-months FU	3	0.31 (− 0.08, 0.69) to 1.00 (− 0.71, 2.71)	Yes	0.229
Change of 6-months FU	3	0.58 (0.28, 0.87) to 1.13 (0.09, 2.18)	Yes	0.485
**Anxiety**				
Change of Post-treatment	11	0.84 (0.48, 1.19) to 1.06 (0.66, 1.46)	Yes	0.045
Change of 6-months FU	4	1.19 (− 0.15, 2.53) to 1.25 (0.08, 2.42)	No	0.249

FU, follow-up.

The Egger test was used to evaluate the publication bias between the studies (Table 3). The included studies that investigated depression and anxiety after follow-up had a significant publication bias (*P* < 0.05). However, the results of the trim-and-fill method suggested that the program did not fill in the fictitious negative results to enhance the symmetry of the funnel plot; further, the meta-analysis results did not change, indicating that the original pooled results were stable. The included studies that investigated the other outcome indicators did not have a significant publication bias (*P* > 0.05).

### Qualitative analysis

Duffy et al.^54^ reported differences in the depression rates between patients with cancer who underwent CBT and TAU at the 6-month follow-up, with the rate in the CBT group decreasing from 68 to 21% and that in the TAU group from 70 to 24%, showing no significant difference between the two groups (*P* > 0.05). Savard et al.^51^ suggested that CBT significantly influenced the depression and anxiety scores at the end of the intervention (*P* < 0.05).

## Discussion

This study analyzed the efficacy of CBT for anxiety and depression across 15 RCTs that included 1979 cancer survivors. The analysis showed that CBT can significantly reduce depression and anxiety in cancer survivors during the intervention period and until 6 months of follow-up, as measured by the depression and anxiety scores, when compared with TAU. The observed effects persisted until the 6-month follow-up, suggesting that CBT provided significant, lasting improvements in depression and anxiety. However, more high-quality RCTs are required to confirm these findings. Additionally, there was no finding that the geographical location, treatment time and treatment form of the included studies affected the heterogeneity.

In a previous meta-analysis and systematic review, with pooled samples of approximately 50,000 long-term cancer survivors, the prevalence of depression and anxiety was 12% and 18%, respectively^16^. Although antidepressants are effective for the treatment of anxiety and depression, they yield poor tolerance, rebound insomnia, and adverse side effects after discontinuation^55^. Given the effects of depression and anxiety on symptom burden and quality of life, evidence supporting effective interventions with minimal side effects and long-term benefits is needed for cancer survivors with anxiety and depression. Evidence from RCTs has indicated that several behavioral approaches, such as mindfulness-based approaches, hypnosis, and self-management strategies, are effective in improving anxiety and depression in cancer survivors^56–58^. However, most studies have been conducted in breast cancer survivors; thus, these interventions need to be further tested in different groups of survivors.

CBT has been demonstrated to be effective in the treatment of depression and anxiety, with well-maintained effects over a 3-month follow-up period^59^. Currently, CBT is recommended as the first-line treatment for depression and anxiety by the National Institute for Health and Care Excellence in the United Kingdom. However, among cancer survivors, the majority of CBT-related studies have focused on those with insomnia^60,61^, with less attention paid to those with depression and anxiety. A recent meta-analysis examined the effect of CBT on the quality of life and psychological health (depression, anxiety, and stress) of patients and survivors of breast cancer. It revealed that CBT is effective in improving the psychological symptoms of both patients and survivors, with meaningful clinical effect sizes^62^. In our study, the beneficial effects of CBT on depression and anxiety in the cancer survivors were maintained until the 6-month follow-up, which suggests the durability of this treatment. Our results are consistent with a previous finding that “individual CBT has short-term effects (< 8 months)” on both depression and anxiety among cancer survivors^63^. Therefore, further research is needed before CBT can be used in the long-term.

It's worth noting that, among the included literatures, several studies were based on internet CBT^39,41,48^. Traditional CBT usually proceeds through face-to-face sessions with a professional in an individual or small-group format and therefore requires significant manpower, time, and cost^64,65^. Internet-based CBT programs is a promising therapeutic alternative that can spread widely within a very short period. They are more accessible and effective than traditional face-to-face interventions in terms of manpower and cost^66^. Internet CBT may provide access to standardized, evidence-based therapy without physical and/or geographical barriers^67^. It has been reported that internet CBT can achieve comparable outcomes to face-to-face CBT for mild to severe anxiety and depression in the general population^68^. Therefore, internet CBT has potential to revolutionize the delivery of CBT, improving the accessibility and availability of CBT content for cancer survivors.

The methodological quality of the included articles herein was moderate; thus, the findings may have the potential to serve as a basis for clinical practice guidelines^69^. Although we applied strict inclusion and exclusion criteria to minimize heterogeneity, there were still high levels of heterogeneity found, which may be attributed to the different methods used to deliver CBT. A subgroup analysis was then used to analyze the potential sources of heterogeneity. The analysis revealed that the geographical location and treatment time were not sources of significant heterogeneity. Importantly, a treatment time of more than 6 weeks was associated with the treatment effect of CBT on both depression and anxiety. Thus, a treatment time of more than 6 weeks is recommended to ensure the efficacy of CBT. Specifically, the subgroup analysis was only performed on the post-treatment changes, since there were fewer than five included studies that conducted 3-, 6-, and 12-month follow-ups. Therefore, the effects of follow-up deserve further attention. Taken together, these findings support recommendations for the use of CBT in survivors of cancer.

### Study strengths and limitations

This study has several strengths. A wide range of databases were searched without restrictions on time scales or language. Strict inclusion and exclusion criteria were used to minimize heterogeneity. The high level of heterogeneity may be attributed to the differences in how CBT was delivered. Study selection and quality assessment were independently completed by two reviewers. The control group was limited to TAU, which can objectively evaluate the intervention effect of CBT. Additionally, the methodological quality of the included studies was moderate, and the control of selection bias, reporting bias, and loss-to-follow-up bias was reasonable. Importantly, although there was significant publication bias for some outcome indicators, the results of both the trim-and-fill method and the one-by-one elimination method suggested the high stability of the pooled results.

This study has some limitations, which might have influenced the results. First, the heterogeneity of the included studies was large, and no significant source of heterogeneity was found in the subgroup analysis. Second, the CBT intervention approaches were inconsistent among the included studies, which is an important source of clinical heterogeneity. Currently, there is no appropriate quantitative method to evaluate the impact on the results of the meta-analysis. Finally, for some outcome indicators, the number of included studies was small, and the sensitivity analysis results were unstable, requiring more large-sample studies to verify the results.

### Clinical implications

Depression and anxiety are highly prevalent concern, affecting cancer survivors and patients. A suite of interventions incorporating cognitive, behavioral, and educational components has been developed for depression and other psychological symptoms^70^. It has been suggested that behavioral interventions are valid for quality of life in cancer patients, and CBT is moderately efficacious for anxiety, depression, and stress symptoms^71,72^. Our study described a statistically significant effect of CBT on depression and distress among cancer survivors, and the results concluded that CBT was an effective intervention in improving depression and distress in cancer survivors during the intervention period and until 6 months of follow-up. Current interventions are often face to face and specialist led. The present mata-analysis included several studies based on internet CBT^39,41,48^, which has potential to revolutionize the delivery of CBT, improving the accessibility and availability of CBT content for cancer survivors. For future studies, it is necessary to address whether intervention effects appear after a continuous intervention.

## Conclusions

This systematic review provided a detailed summary of the evidence on the effect of CBT interventions on depression and anxiety among cancer survivors and evaluated dynamic data at 3–12 months of follow-up. Compared with TAU, CBT significantly improved the depression and anxiety scores of the cancer survivors, and this improvement was maintained until the 6-month follow-up. It is recommended that more large-sample, high-quality RCTs be conducted for verification.

## Supplementary Information

Supplementary Information.

## Data Availability

All data generated or analyzed in this study are included in this published article and its supplementary information files.
