# Supplementary material for: Cognitive behavioral therapy for anxiety and depression in cancer survivors: a meta-analysis
Source: Sci Rep. 2022 Dec 12;12:21466. doi: 10.1038/s41598-022-25068-7 (PMC9744858; doi:10.1038/s41598-022-25068-7)

**Supplementary Table 1** PubMed search steps and results (The retrieval time: 20220523)

| Search | Query | Items |
| --- | --- | --- |
| #1 | "neoplasms"[MeSH Terms] | 3687931 |
| #2 | "neoplasms"[tiab] OR "cancer"[tiab] OR "cancers"[tiab] OR "tumour"[tiab] OR "tumor"[tiab] OR "tumors"[tiab] OR "tumours"[tiab] | 3273153 |
| #3 | #1 OR #2 | 4623139 |
| #4 | ("cognitive behavioral therapy"[MeSH Terms] OR "cognitive behavioral therapy"[tiab] OR Behavioral Therapies, Cognitive[tiab] OR Behavioral Therapy, Cognitive[tiab] OR Cognitive Behavioral Therapies[tiab] OR Therapies, Cognitive Behavioral[tiab] OR Therapy, Cognitive Behavioral[tiab] OR Psychotherapy, Cognitive[tiab] OR Therapy, Cognitive[tiab] OR Cognitive Therapies[tiab] OR Therapies, Cognitive[tiab] OR Cognitive Therapy[tiab] OR Cognitive Behaviour Therapy[tiab] OR Behaviour Therapies, Cognitive[tiab] OR Behaviour Therapy, Cognitive[tiab] OR Cognitive Behaviour Therapies[tiab] OR Therapies, Cognitive Behaviour[tiab] OR Therapy, Cognitive Behaviour[tiab] OR Cognitive Psychotherapy[tiab] OR Cognitive Psychotherapies[tiab] OR Psychotherapies, Cognitive[tiab] OR Cognition Therapy[tiab] OR Cognition Therapies[tiab] OR Therapies, Cognition[tiab] OR Therapy, Cognitive Behavior[tiab] OR Behavior Therapies, Cognitive[tiab] OR Cognitive Behavior Therapies[tiab] OR Therapies, Cognitive Behavior[tiab] OR Therapy, Cognition[tiab] OR Behavior Therapy, Cognitive[tiab] OR Cognitive Behavior Therapy[tiab]) | 68999 |
| #5 | "anxiety"[MeSH Terms] OR "anxiety"[tiab] OR "anxieties"[tiab] | 263524 |
| #6 | "depressed"[tiab] OR "depression"[MeSH Terms] OR "depression"[tiab] OR "depressions"[tiab] OR "depressive disorder"[MeSH Terms] OR "depressive disorder"[tiab] OR "depressivity"[tiab] OR "depressively"[tiab] OR "depressiveness"[tiab] OR "depressives"[tiab] | 522648 |
| #7 | #5 OR #6 | 662433 |
| #8 | #3 AND #4 AND #7 | 1019 |

**Supplementary Figure 1.** Methodological quality assessment results of the included studies.


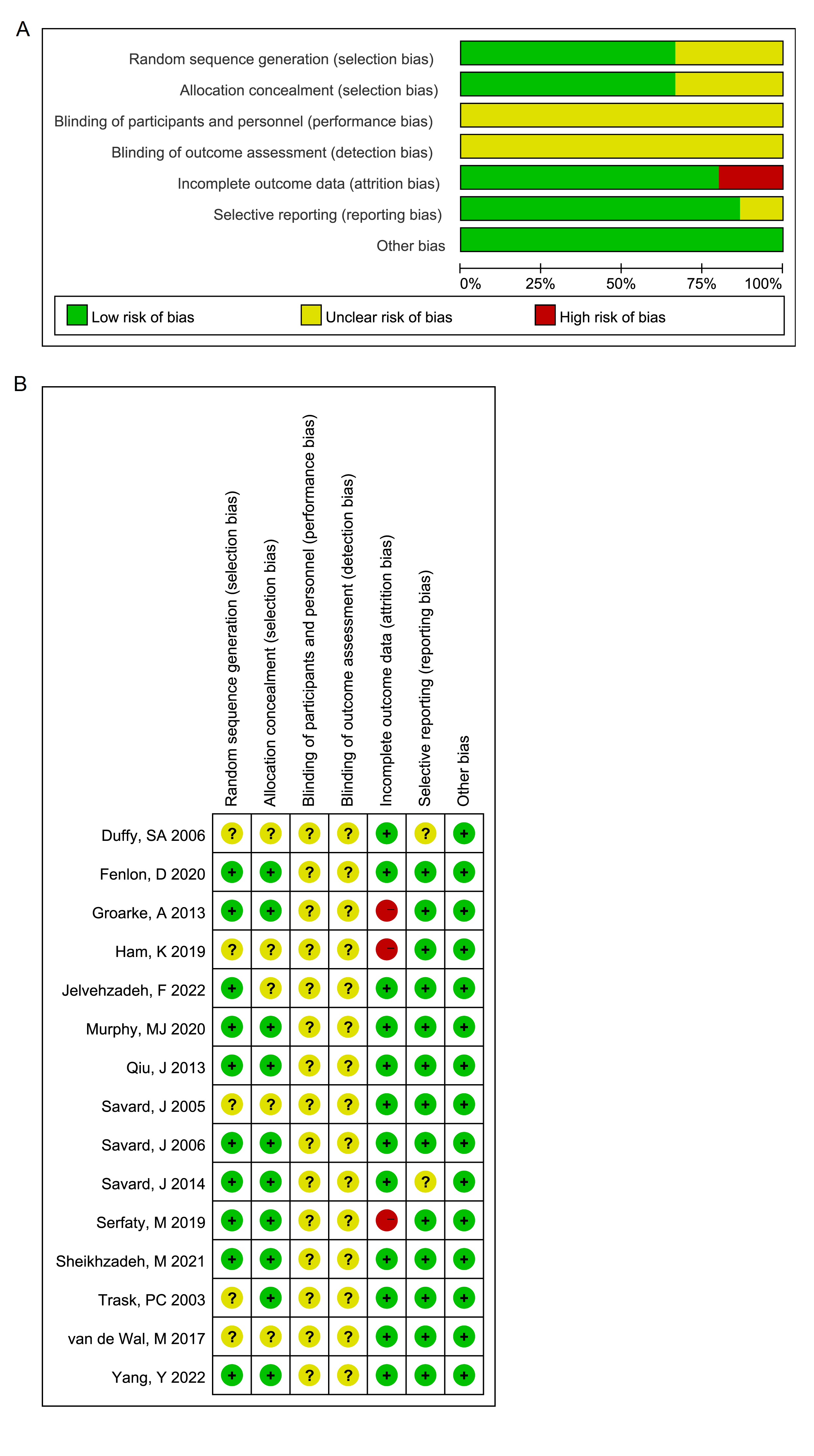
**Supplementary Figure 2.** Forest plot of the subgroup analyses of the depression scores. The subgroup analysis according to the geographical location (A) and the treatment time (B) and the treatment form (C).


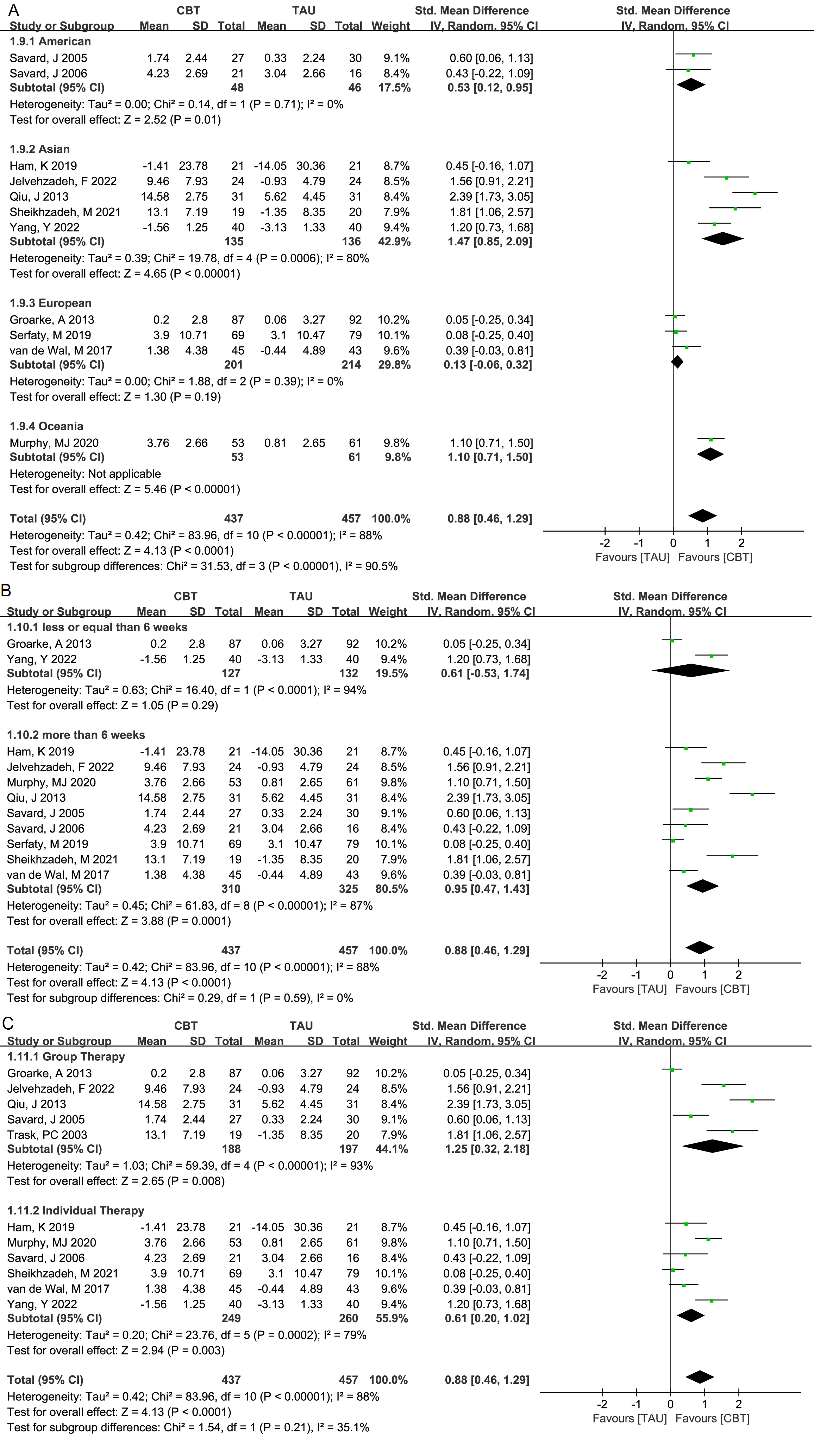


**Supplementary Figure 3.** Forest plot of the subgroup analyses of the anxiety scores. The subgroup analysis according to the geographical location (A) and the treatment time (B) and the treatment form (C).


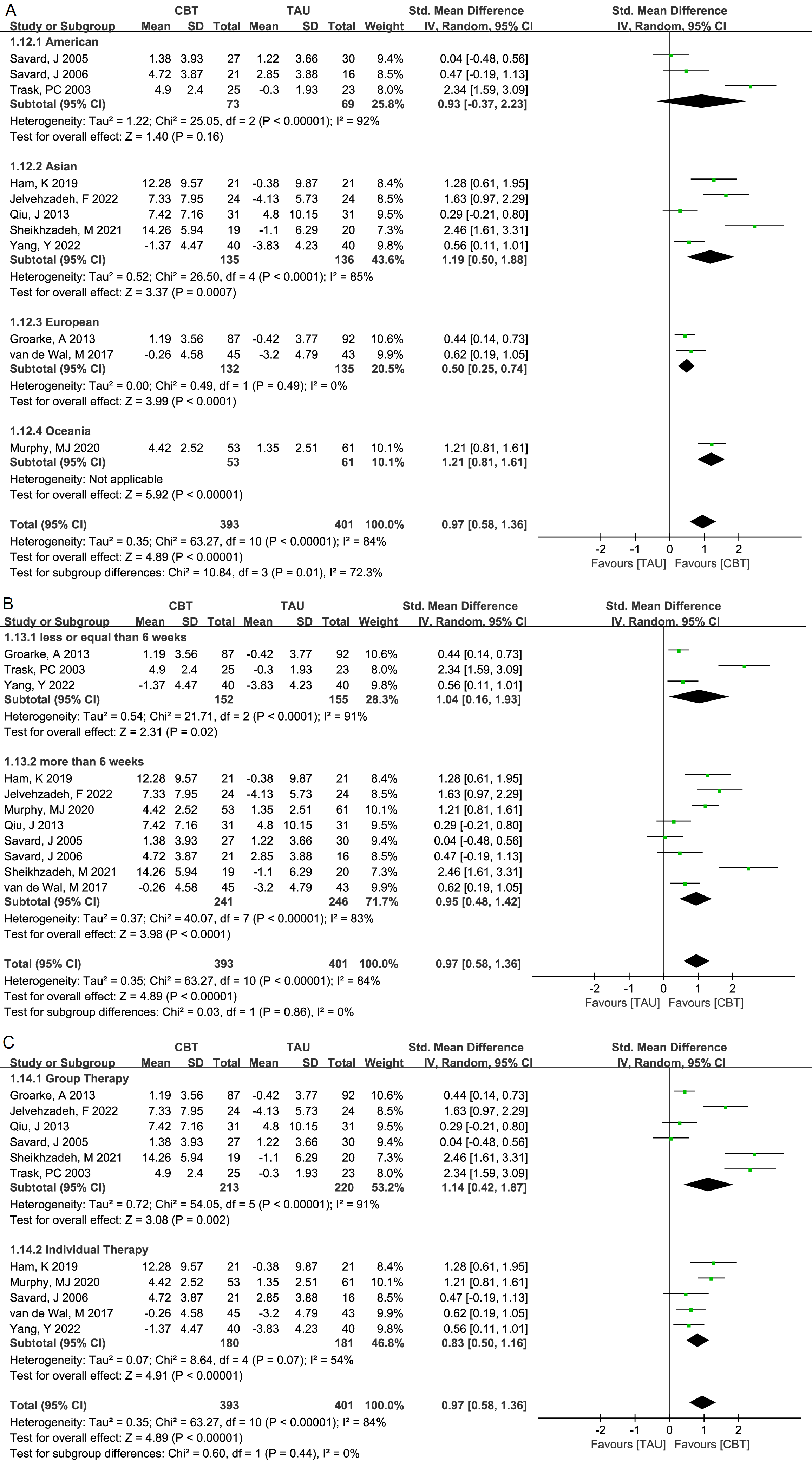

Supplement: Supplementary file 1 — Supplementary Information. [file 41598_2022_25068_MOESM1_ESM.docx]
